# Basic Psychological Needs at Work: Their Relationship with Psychological Well-Being and Healthy Organisational Outcomes with a Gender Perspective

**DOI:** 10.3390/ijerph19053103

**Published:** 2022-03-06

**Authors:** Alexandra Gil-Flórez, Susana Llorens, Hedy Acosta-Antognoni, Marisa Salanova

**Affiliations:** 1 WANT Research Team, Universitat Jaume I, 12071 Castelló de la Plana, Castelló, Spain; gilalexandra@gmail.com (A.G.-F.); salanova@uji.es (M.S.); 2 Faculty of Psychology, University of Talca, Talca 3480094, Chile; hacosta@utalca.cl

**Keywords:** Basic Psychological Needs, engagement, happiness, Healthy Organisational Outcomes, gender perspective

## Abstract

The aim of the study is to evaluate the mediating role of psychological well-being between the satisfaction/frustration of Basic Psychological Needs (BPN) and Healthy Organisational Outcomes, with a gender perspective. In a sample of 565 workers (65% women, response rate 72%) from two Colombian food companies, using Multigroup Structural Equation Models to test the invariance among gender, the study hypotheses were partially supported. The results show that: (1) psychological well-being fully mediates the relationship between BPN satisfaction and Healthy Organisational Outcomes; and (2) only work engagement mediates the relationship between BPN frustration and Healthy Organisational Outcomes. Specifically, people (women and men) whose basic needs are satisfied experience greater well-being and better Healthy Organisational Outcomes. In contrast, people whose BPN are frustrated experience lower levels of work engagement, which, in turn, influences their Healthy Organisational Outcomes. However, structural differences were observed among the variables, attending to gender, when invariance model grouping by gender was tested. Furthermore, ANOVA by gender found significant differences in the autonomy dimension of frustration and organisational affective commitment, in favour of men. The present study contributes to the scarce research on the role of BPN levels as a relevant driver in the development of psychological well-being and Healthy Organisational Outcomes.

## 1. Introduction

The Self-Determination Theory (SDT), proposed by Deci and Ryan in the 1980s, has received considerable attention, especially from Positive Psychology [1]. This theory raises the importance of the individual as an active human being in search of the definition of his/her personality through the satisfaction of needs [2]. Gagné and Deci [3], in a review of empirical studies related to SDT, point out that supervisors’ support generates high levels of satisfaction with competence, relationships, and autonomy which, in turn, leads to psychosocial well-being and better organisational results, such as higher job satisfaction, organisational commitment, improved performance evaluations, persistence, acceptance of organisational change, and greater psychological adjustment. The SDT proposes that a person has three basic needs: autonomy, relationships, and competence, all of which motivate their actions to satisfy them. Research on the impact of Basic Psychological Needs (BPN) at work is still scarce. However, they have been studied in sports research [4,5,6] and education [7,8]. Along the same line, Vansteenkiste et al. [9] highlighted that psychological need is an essential nutrient for the adjustment, integrity, and growth of individuals. This allows the renaissance of the study of Basic Psychological Needs (BPN) in the present millennium, to expand these studies and validate new areas of application, such as organisation. Therefore, the first aim of the present study is to integrate the investigation of BPN and psychological well-being into an organisational context.

Regarding psychological well-being, in 1999 Martin Seligman proposed the Positive Psychology approach, oriented towards the scientific study of human strengths and qualities that generate positive and effective results, taking into account their state of mind and emotions, changing the person’s thought structure, and focusing on the bright side of life [10,11]. From Positive Psychology, efforts are being made to investigate and build models and experiences that develop well-being (i.e., happiness, work engagement), so that people can adapt to different environments and crises, with organisations being important change managers that promote the well-being and health of their workers [12,13].

In the present study, two psychological well-being variables are considered: (1) happiness and (2) work engagement, because they integrate non-work and work well-being. Happiness (scientifically known as psychological well-being) refers to a state of mind that leads to experiencing joy, pleasure, ecstasy, comfort, and other positive emotions [14]. Work engagement (WE) is understood as a positive mental state of accomplishment at work that is characterised by vigour, dedication, and absorption [15]. From a psychosocial perspective, WE stimulates employees’ motivation processes that begin with greater availability of resources to perform their work, more guidance and training, and self-regulation of their behaviour [15,16]. In addition, work engagement is a key element in the development of healthy and resilient organisations [15,16,17].

An Occupational Health Psychology model that allows a comprehensive analysis perspective [18] is the Healthy and Resilient Organisations Model (HERO, Healthy & Resilient Organisations). According to this model, HEROs are defined as ‘organisations that make systematic, planned, and proactive efforts to improve the health of employees using good practices’ [18,19,20]. Through Structural Equation Models (SEM) in a sample of 1484 employees, 303 teams and their supervisors, 43 companies, and 2098 clients/users, the HERO Model was validated, and integrates three key elements: (1) healthy organisational practices and resources (e.g., work-life balance and leadership), (2) healthy employees (e.g., work engagement), and (3) Healthy Organisational Outcomes (e.g., in-role and extra-role performance). These three elements interact with each other and allow the company to develop in a changing environment and prepare to face a crisis successfully [18,19,20]. Research on the HERO Model provides strong empirical evidence showing that the practices and resources implemented by organisations will have an impact on positive results as long as workers feel cared for and experience well-being (i.e., happiness, WE). Despite this model’s relevance, the impact of BPN as personal resources in the development of motivation processes and their role in developing a HERO have not been tested.

Therefore, the present study aims to evaluate the mediating role of psychological well-being between the satisfaction/frustration of Basic Psychological Needs (BPN) and Healthy Organisational Outcomes from a gender perspective, using data obtained from two companies within the Colombian industrial sector. More specifically, the novelty of the present study is to examine the consequences of BPN (including satisfaction and frustration dimensions) on well-being (engagement and happiness) and Healthy Organisational Outcomes, considering the invariance of gender perspective. In this way, we propose that BPN acts as a positive driver in the development of well-being and Healthy Organisational Outcomes, regardless of gender, in two Colombian food companies.

## 2. Theoretical Framework

### 2.1. Basic Psychological Needs (BPN) and Well-Being

The SDT, published by Edward Deci and Richard Ryan [21,22,23], links human motivation to personality and optimal functioning. It states that people are motivated to change and grow by obtaining BPN, which form them as self-determined and motivated individuals. This study’s central focus is on the BPN Theory, which deals with the energisation of behaviour. Although empirical research in the organisational context is incipient, the theory focuses on behaviour and, in turn, the search for satisfaction of the BPN of autonomy, relatedness, and competence. Autonomy corresponds to an experience of integration and freedom [8], through which individuals feel that they can choose their actions and accept the consequences, control their behaviour and goals, be effective, and accept challenges. Relatedness generates belonging and fosters healthy social relationships, thus making people sensitive, receptive, and capable of empathising with others. They connect with these feelings and respond to them, even at the affective level. Finally, competence refers to feelings of effectiveness and efficacy in the role one plays when interacting with the social environment [22].

These needs are fundamental to the development of psychological health and personal well-being, and they are described as innate and universal. In turn, the theory also includes the frustration that arises from not fulfilling these needs. In this sense, both dimensions of BPN are crucial in understanding the fulfilment of these needs [21].

Regarding BPN frustration, Bernárdez [24] carried out an investigation in 67 schools in Argentina and showed that BPN frustration is linked to high levels of work stress, absenteeism, and physical and psychological discomfort. This observation opens the door to empirical research on the impact of integrating satisfaction and frustration of BPNs in the search for well-being (i.e., happiness and work engagement) in organisations and their relationship with Healthy Organisational Outcomes (i.e., performance and commitment).

Regarding the relationship between BPN and well-being, studies have found that, when these innate needs are satisfied, they produce an increase in self-motivation, mental health, and well-being [25,26,27]. For example, Hernández-Andreo et al. [6], in a sample of 97 physical education students, showed that one way of experiencing happiness was linked to the achievement of set goals. In other research, they found that the well-being experienced at work could influence how workers felt in other vital areas, improving their relationships with management and decreasing their anxiety levels [26,27,28,29]. Similar relationships have been obtained regarding work engagement. Specifically, work engagement (WE) refers to a particular way of relating to work or a task [15,30,31,32]. It has been shown to be a robust indicator of well-being in the workplace in Europe [15,31,32]. Although there are approaches to the study of WE in Colombia [33,34], it is relevant to demonstrate the existence of a significant and positive role of WE as a mediator between BPN and Healthy Organisational Outcomes. For example, Blasco-Giner and Meneghel [35], in a sample of 195 employees, showed that engaged workers experience a balance between challenges and skills, and, when this psychological state persists over time, it is linked to feelings of belonging to an organisation. WE increases energy levels and improves adaptation to unexpected events or situations. It makes it possible to respond to these events with a greater sense of self-efficacy and resilience, with enthusiasm, and without measuring time, as they are immersed in their activity because they enjoy it [35]. So far, the evidence proposes a direct and significant relationship between the three needs (i.e., autonomy, relatedness, and competence) and WE (i.e., vigour, dedication, and absorption) in the field of education and sports [6,25]. The present study will focus on investigating these relationships in an organisational context.

### 2.2. Well-Being and Healthy Organisational Outcomes

Based on previous research, there is evidence in favour of the relationship between BPN, well-being, and Healthy Organisational Outcomes (i.e., job performance and organisational affective commitment). Job performance has been integrated into studies for more than 100 years, and debates and research into it continue to this day (e.g., [36]). Borman and Motowidlo [37] define performance as a value that contributes directly towards achieving the organisation’s objectives, due to the behaviour of individuals in work teams. Thus, the work by Goodman and Svyantek [38] is considered relevant because they introduce two dimensions of job performance: in-role and extra-role. On the one hand, the in-role dimension is defined as those activities that contribute directly or indirectly to the technical base of the organisation, and these can vary among different jobs within the organisation. Sotelo [39] proposes that self-determination is a key element in employees’ motivation to carry out their work. His results show that the need for autonomy in performing tasks, recognition, and the quality of relationships at work are found to be closely linked, with all the dimensions showing more than 80% acceptance by the worker. On the other hand, the extra-role dimension is defined as those activities that are not formally part of the job and that employees perform voluntarily, even though they involve additional effort beyond satisfactorily completing their usual activities [40,41]. On the other hand, commitment has to do with the ways of committing to the organisation [31,32,42]. In 1984, Meyer and Allen [43] suggested that commitment has three facets: affective (workers’ degree of identification), continuance (employees’ need to continue in the position), and normative (perceived obligation to remain in the institution). Each facet was composed of different elements, such as rules, norms, and satisfaction of needs [44]. In an organisational context, the affective commitment dimension is the one that has received the most attention. It is understood as the desire to continue to belong to the organisation and responds to the emotional disposition of affectively bonding with it [45].

Regarding the mediating role of psychological well-being, Wright and Doherty [46] point out that the decisive element in commitment is psychological well-being. This presents different challenges for organisations, because they need workers who act in accordance with the proposed objectives, improve the tasks, and work effectively and efficiently with the work team, ultimately optimising outcomes [47,48]. Similarly, Roa and Avendaño [49], in a sample of 412 employees from six Colombian companies within different sectors, found a direct relationship between happiness and performance, showing that the individual’s satisfaction and well-being influences performance. In addition, regarding the engagement relationship, Cruz-Ortiz et al. [40], applying SEM in a sample of 519 workers from 58 work teams belonging to 12 Spanish SMEs, showed that work engagement is linked to better performance at work. In the same way, well-being improves the service climate and employees’ performance, positive affect, collective effectiveness, and team performance [50].

### 2.3. Basic Psychological Needs from a Gender Perspective

Previous literature has evidence in favour of the invariance among gender of Self-determination Theory (SDT) in different fields such as sports and education (e.g., [51]). Specifically, Guérin et al. [52] stressed insignificant differences across gender in exercise results, particularly in relation to SDT. On the other hand, in an applied study [53] on a sample of 888 master’s students (461 women, 427 men), an analysis was carried out on whether or not the perceptions of autonomy and gender-specific competence affected their decisions about their enrolment in STEM subjects (science, technology, engineering, and mathematics). The results revealed that male students perceive themselves as having more self-efficacy and leadership than women, who showed low perceptions of their competence and uncertainty with regards to the future, and, therefore, less motivation [53]. Another study, carried out over eight months among 85 students, evaluated a program based on the teaching of Personal and Social Responsibility, including Basic Psychological Needs, motivation, life satisfaction, and gender differences. The results pointed out that, in general, there is improvement in terms of personal responsibility, and, in the case of female students, improvements are also evident in BPN and intrinsic motivation [54,55].

Now, the theory of self-determination has evolved, and has been extended to include research within the work organisation, since all employees (women and men) have three basic needs (competence, autonomy, and relationship), which, when satisfied, promote motivation, performance, and well-being [56,57]. Despite the theory’s relevance, the literature on the gender perspective and its effect on Basic Psychological Needs in the workplace is a novelty, and features in the present study.

### 2.4. The Current Study

The present study aims to evaluate the mediating role of psychological well-being (i.e., happiness and work engagement) between the satisfaction/frustration of Basic Psychological Needs (BPN) (i.e., autonomy, relatedness, and competence) and Healthy Organisational Outcomes (i.e., in-role and extra-role job performance and organisational affective commitment) from a gender perspective, using data obtained from two companies within the Colombian industrial sector. Specifically, we examine the consequences of BPN (including satisfaction and frustration dimensions) on well-being (engagement and happiness) and Healthy Organisational Outcomes, taking into account the invariance of gender perspective. In this way, we propose that BPN acts as a positive driver in the development of well-being and Healthy Organisational Outcomes in two Colombian food companies, regardless of gender.

**Figure 1:**
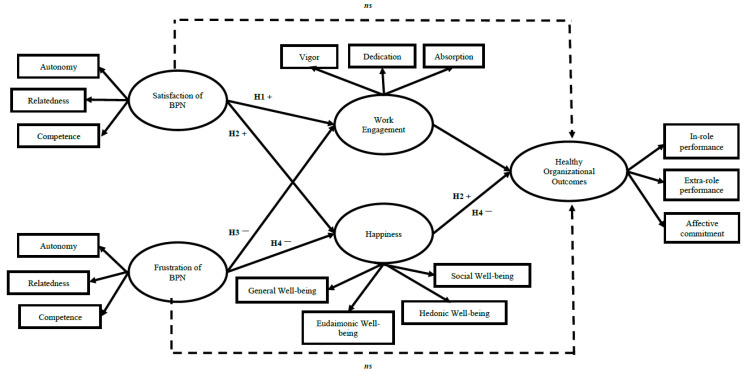
Research model.

Based on the Self-Determination Theory [56] DECI OLAFSEN, we could expect that psychological well-being (i.e., work engagement and happiness) is expected to fully mediate the relationship between BPN and Healthy Organisational Outcomes (in-role and extra-role performance and organisational affective commitment) in women and men. Specifically, it is expected that (see Figure 1):

**Hypothesis** **1.** *Work engagement positively mediates the relationship between BPN satisfaction and Healthy Organisational Outcomes, regardless of gender. That is, we expect that BPN satisfaction will be positively linked to work engagement, which, in turn, will be positively linked to Healthy Organisational Outcomes, regardless of gender (Hypothesis 1).*

**Hypothesis** **2.** *Happiness positively mediates the relationship between BPN satisfaction and Healthy Organisational Outcomes, regardless of gender. That is, we expect that BPN satisfaction will be positively linked to happiness, which, in turn, will be positively linked to Healthy Organisational Outcomes, regardless of gender (Hypothesis 2).*

**Hypothesis** **3.** *Work engagement negatively mediates the relationship between BPN frustration and Healthy Organisational Outcomes, regardless of gender. That is, we expect that BPN frustration will be negatively linked to work engagement, which, in turn, will be negatively linked to Healthy Organisational Outcomes, regardless of gender (Hypothesis 3).*

**Hypothesis** **4.** *Finally, happiness negatively mediates the relationship between BPN frustration and Healthy Organisational Outcomes, regardless of gender. That is, we expect that BPN frustration will be negatively linked to happiness, which, in turn, will be negatively linked to Healthy Organisational Outcomes, regardless of gender (Hypothesis 4).*

## 3. Materials and Methods

### 3.1. Participants

The sample consisted of 565 workers who belonged to a specific sample: two Colombian food production organisations within the industrial sector, with offices in Barranquilla (6.4%), Bogotá (87.8%), and Ibagué (5.8%). In addition, 65% (369) were women, 34% (192) were men, and 1% (4) did not provide this information.

The procedure in both organisations began with the presentation of the study, making special mention of the benefits of participating in said study (the first study in Colombia). Once authorisation had been obtained by the CEO of each company to participate in said research, the meetings began. Three meetings were held with the CEOs and key agents of both organisations. The first meeting aimed to present the project to the CEOs, and the second to the key agents, including managers and the Human Resources department. In the third meeting, the logistics of the information gathering process were outlined. The data was collected between the years 2018 and 2019. The questionnaire was carried out during the workday. The Human Resources department in both companies arranged a place and groups so that the workers could fill in the questionnaires in shifts. Once the workers were gathered, the research objective was explained to them, and those who wished to participate voluntarily were asked to sign the informed consent. A researcher, who answered questions and collected the questionnaires at the end of the process, led the sessions. Employees completed a self-report questionnaire (pencil and paper) that took approximately 40 min to fill out. Confidentiality was guaranteed. The Ethics Committee of Jaume I University approved the study (CD/62/2020). With regards to the response rate: it was 87.5% (63/72) in the first organisation and 89% (634/712) in the second. Only complete response questionnaires were considered in this research, corresponding to 565 participants—a response rate of 72%. This sample is representative for computing SEM analyses. Specifically, the results show that, for a power of 0.80, df = 90, we need a sample of 142. In the manuscript, our total sample consists of 565, including 369 women and 192 men [58].

### 3.2. Measurement Instruments

Satisfaction and Frustration of *Basic Psychological Needs (BPN)* were evaluated with the Spanish version of the questionnaire by Chen et al. [59]. It contains 24 items distributed in two dimensions (BPN satisfaction and BPN frustration), rated on a Likert-type scale with seven points (0 ‘totally false’ to 6 ‘totally true’). Both dimensions were tested together, since they do not imply a positive or negative aspect of the seam continuum [22]. Specifically, BPN satisfaction was evaluated using three scales (12 items): (1) satisfaction with autonomy (four items: e.g., ‘I feel that I have freedom and choice in the things I undertake’; alpha = 0.70); (2) satisfaction in relationships with others (four items; e.g., ‘I feel that people I care about care about me’; alpha = 0.70); and (3) satisfaction with competence (four items; e.g., ‘I feel confident I can do things well’; alpha = 0.82). Second, BPN frustration was evaluated using three scales (12 items): (1) frustration of autonomy (four items; e.g., ‘I feel that most of the things I do, I only do them because “I have to do them”’; alpha = 0.71); (2) frustration in interpersonal relationships (four items; e.g., ‘I feel excluded from the group I want to belong to’; alpha = 0.71); and (3) competence frustration (four items; e.g., ‘I have serious doubts about whether I can do things well’; alpha = 0.70).

*Work Engagement* (WE) was evaluated using the Spanish version [15] of the Utrecht Work Engagement Scale (UWES) (rated from 0 ‘never’ to 6 ‘always’), with nine items in three dimensions (1) vigour (three items; e.g., ‘During work, I feel full of energy’; alpha = 0.84); (2) dedication (three items; e.g., ‘I’m excited about my work’; alpha = 0.81); and (3) absorption (three items; e.g., ‘Time flies by when I’m working’; alpha = 0.72).

*Happiness* was evaluated using the Pemberton Happiness Index (PHI), validated in Spanish by Hervás and Vázquez [60]. The scale (alpha = 0.83) was composed of 11 items, nested in four dimensions and rated from 0 ‘strongly disagree’ to 10 ‘strongly agree’. The four dimensions were: (1) general well-being (two items: ‘I feel satisfied with my life’ and ‘I have the necessary energy to accomplish my daily tasks well’; r = 0.51***); (2) eudemonic well-being (six items; e.g., ‘I feel happy with the way I am’; alpha = 0.84); (3) hedonic well-being (two items: ‘I enjoy many little things every day’ and ‘I have a lot of bad moments in my day to day life (reverse)’; r = 0.24*); and (4) social well-being (one item; ‘I think I live in a society that lets me fully realise my potential’).

*Healthy Organisational Outcomes* were evaluated with the dimensions of in-role and extra-role job performance and organisational affective commitment through the HERO questionnaire [20], with items rated on a 7-point Likert scale (0 ‘strongly disagree’ to 6 ‘strongly agree’). Specifically, job performance was evaluated with the Goodman and Svyantek [38] performance questionnaire, which was composed of six items distributed in two dimensions: (1) in-role (three items; e.g., ‘achievement of the work objectives’; alpha = 0.82) and (2) extra-role (three items; e.g.,’ I help colleagues with their work when they have to be absent ‘; alpha = 0.78). On the other hand, organisational affective commitment was evaluated with three items from the Allen and Meyer scale [61]. An example of an item is: ‘The organisation’s problems are our problems’ (alpha = 0.79).

### 3.3. Data Analyses

First, descriptive analyses were calculated through the individual database, using the SPSS and AMOS programs from the IBM package (25.0 version). Second, an analysis of variance (ANOVA) was carried out to evaluate significant differences based on the sociodemographic variables (i.e., sex, age, type of contract, and tenure). Third, the Harman one-factor test was performed to assess common variance bias [62].

Subsequently, the Structural Equations Model was used, with the AMOS 22.0 statistical program, in order to test the relationship between BPN (i.e., satisfaction and frustration), WE (i.e., vigour, dedication and absorption), happiness, and Healthy Organisational Outcomes (in-role work performance, extra-role work performance, and affective organisational commitment). In addition, a double mediation model, M1, was evaluated in which well-being (work engagement and happiness) mediates the relationship between BPN and Healthy Organisational Outcomes (in-role job performance, extra-role job performance, and organisational affective commitment) following the recommendation of Preacher and Hayes [63]. In addition, an Alternative Model, MA, was tested, in which BPNs mediate the relationship between WE and happiness and Healthy Organisational Outcomes. Additionally, an invariance model by gender was conducted by Multigroup SEM in order to test the significant differences in the relationship between BPN (i.e., satisfaction and frustration), WE (i.e., vigour, dedication and absorption), happiness, and Healthy Organisational Outcomes (in-role work performance, extra-role work performance, and affective organisational commitment).

The estimation method used was maximum probability. Three absolute indices were evaluated to show the goodness of fit of the models: the χ^2^ statistic, the Root Mean Square Error of Approximation (RMSEA), and the absolute goodness of fit index (CMIN/DF) [64]. The χ^2^ is sensitive to the sample size, and so the use of relative indices is recommended to evaluate the goodness of fit of the models. Three relative indices of goodness of fit of the models were evaluated: (1) CFI (Comparative Fit Index), (2) NFI (Normed Fit Index), and (3) IFI (Incremental Fit Index) [60,61,62]. Subsequently, the AIC index (Akaike Information Criterion) was used to compare non-nested models, as well as the HOELTER index, which corresponds to the sample size necessary to accept the Chi-Square test [65].

## 4. Results

### 4.1. Demographic and Descriptive Analyses and ANOVA

Table 1 shows demographic information regarding the sample within the Colombian food industry. Table 2 shows the means, standard deviations (generally and by gender), Cronbach’s alpha, and intercorrelations of all the variables included in the study (N = 565), i.e., BPN (i.e., satisfaction and frustration), work engagement (i.e., vigour, dedication, and absorption), happiness, and Healthy Organisational Outcomes (in-role job performance, extra-role job performance, and organisational affective commitment), using the PASW 22.0 program. In addition, analyses of normality were performed. The KMO and Bartlett tests were performed, indicating that the null hypothesis was confirmed, meaning that the data distribution was normal (KMO = 0.85, Bartlett = 3105.01, *p* = 0.6).The results in our specific sample within the Colombian food industry show, as expected, that: (1) BPN satisfaction correlates positively and significantly with work engagement, happiness, and Healthy Organisational Outcomes (i.e., in-role performance, extra-role performance, and affective organisational commitment); (2) BPN frustration correlates negatively and significantly with work engagement, happiness, and Healthy Organisational Outcomes (i.e., in-role performance, extra-role performance, and organisational affective commitment); and (3) psychological well-being (i.e., work engagement and happiness) positively correlates with Healthy Organisational Outcomes. The internal consistency of all the scales meets the criterion of 0.70 [66] NUNNALY. The Harman single factor test showed, as expected, a poor fit to the data χ^2^ (105) = 1177.60, RMSEA = 0.16, CFI = 0.52, TLI = 0.380, IFI = 0.53, IFI = 0.50. Following the recommendations of Podsakoff, McKenzie, and Podsakoff [67], the questionnaire also had different sections and different instructions. Therefore, common variance bias does not pose a problem in the study data.

Third, the ANOVA showed that: (1) in terms of gender, there were no significant differences between the study variables, except in the autonomy dimension of frustration of BPN, F = 4.90; *p* < 0.03, and organisational affective commitment, F = 6.62, *p* < 0.01, in favour of men; (2) in terms of age and (3) tenure, there were no significant differences between the study variables; and (4) in terms of type of contract, there were no significant differences between the study variables, except happiness in its hedonic well-being dimension (F = 6.06, *p* < 0.001).

### 4.2. Model Fit: Structural Equation Modelling

With the individual database (N = 565), five latent variables were used: (1) BPN satisfaction was composed of three indicators: autonomy satisfaction, relatedness satisfaction, and competence satisfaction; (2) BPN frustration was composed of three indicators: autonomy frustration, relatedness frustration, and competence frustration; (3) Work engagement was composed of three indicators: vigour, dedication, and absorption; (4) Happiness was composed of four indicators: general well-being, eudemonic well-being, hedonic well-being, and social well-being; and, (5) Healthy Organisational Outcomes was composed of three indicators: in-role job performance, extra-role job performance, and organisational affective commitment.

Table 3 shows the results of the structural equation models (one round was performed). The findings of these analyses indicate that, in this specific sample within the Colombian food industry, in the proposed mediation model (M1), work engagement (i.e., vigour, dedication, and absorption) and happiness (i.e., general well-being, eudemonic well-being, hedonic well-being, and social well-being) partially mediate the relationship between BPN (i.e., satisfaction and frustration) and Healthy Organisational Outcomes (i.e., in-role job performance, extra-role job performance, and organisational affective commitment). The model tested fits the data well, χ^2^ (94) = 300.07, χ^2^/df = 3.19, RMSEA = 0.07, CFI = 0.91, NFI = 0.87, IFI = 0.91, AIC = 416.08, HOELTER = 156 (0.05) and 170 (0.01). Regarding the relationship between the variables, the results show that BPN satisfaction (i.e., autonomy, relatedness, and competence) (1) is positively and significantly related to work engagement (i.e., vigour, dedication, and absorption), β = 0.15, *p* < 0.05, and happiness (i.e., general well-being, eudemonic well-being, hedonic well-being, and social well-being), β = 0.38, *p* < 0.01; (2) but it is not significantly related to Healthy Organisational Outcomes (i.e., in-role job performance, extra-role job performance, and affective organisational commitment), β = 0.09, *p* = 0.31.

Regarding BPN frustration (i.e., autonomy, relatedness, and competence), the results show (1) a negative and significant relationship with work engagement (i.e., vigour, dedication, and absorption), β = −0.23, *p* < 0.05; and (2) non-significant relationships with happiness (i.e., general well-being, eudemonic well-being, hedonic well-being, and social well-being), β = −0.02, *p* = 0.48, and Healthy Organisational Outcomes (i.e., in-role job performance, extra-role job performance, and organisational affective commitment), β = 0.06, *p* = 0.47.

**Figure 2:**
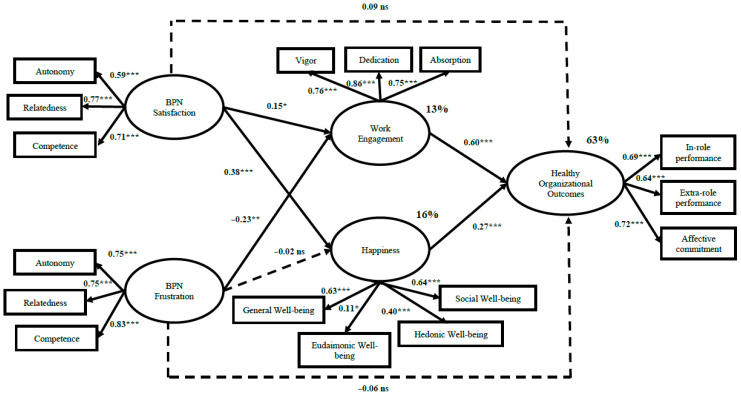
Final Model: Structural model of BPN, well-being, and Healthy Organisational Outcomes, regardless of gender (N = 565). Notes: * *p* < 0.05; ** *p* < 0.01, *** *p* < 0.001.

In the same way, work engagement and happiness are positively and significantly linked to Healthy Organisational Outcomes, β = 0.60, *p* < 0.01; β = 0.27, *p* < 0.01, respectively. It is interesting to note that BPNs (i.e., satisfaction and frustration) explain 13% (*p* < 0.01) of work engagement and 16% (*p* < 0.01) of happiness. In addition, work engagement and happiness explain 63% (*p* < 0.01) of Healthy Organisational Outcomes (i.e., in-role job performance, extra-role job performance, and organisational affective commitment). See Figure 2.

Regarding the Alternative Model (MA), the results show a poor data fit with regards to its absolute and relative indicators, χ^2^ (96) = 467.31, χ^2^/df = 4.87, RMSEA = 0.09, CFI = 0.83, NFI = 0.80, IFI = 0.84, AIC = 579.31, HOELTER = 102 (0.05) and 112 (0.01), indicating that psychological well-being mediates the relationship between satisfaction/frustration of BPN and Healthy Organisational Outcomes.

### 4.3. Model Fit: Multigroup Structural Equation Modelling by Gender

**Figure 3:**
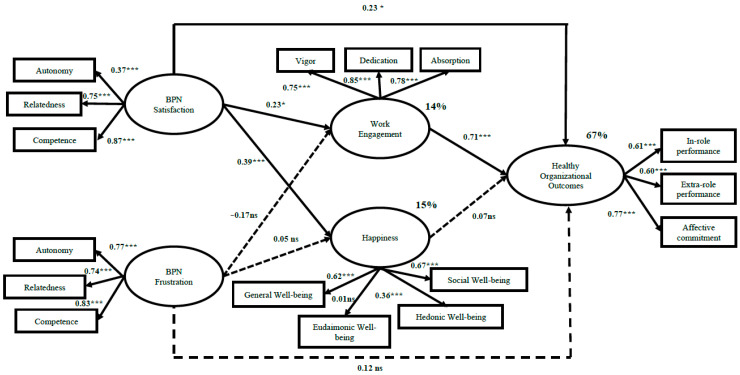
Final Model: Structural model of BPN, well-being, and Healthy Organisational Outcomes in women (N = 262). Notes: * *p* < 0.05; ** *p* < 0.01, *** *p* < 0.001.

Regarding the invariance model grouping by gender (N = 262 women; N = 133 men), the Structural Equation Model in this sample within the Colombian food industry shows that: (1) in the women’s group model (see Figure 3), work engagement (i.e., vigour, dedication, and absorption) partially mediates the relationship between BPN satisfaction (i.e., autonomy, relatedness, and competence) and Healthy Organisational Outcomes (i.e., in-role job performance, extra-role job performance, and organisational affective commitment). Furthermore, BPN satisfaction has a positive and significant relationship with happiness (i.e., general well-being, eudemonic well-being, hedonic well-being, and social well-being). However, BPN frustration (i.e., autonomy, relatedness, and competence) has no significant relationship with work engagement, happiness, or Healthy Organisational Outcomes. The results show that BPN satisfaction (i.e., autonomy, relatedness, and competence) is positively and significantly related to work engagement (i.e., vigour, dedication, and absorption), β = 0.23, *p* < 0.05, and happiness (i.e., general well-being, eudemonic well-being, hedonic well-being, and social well-being), β = 0.71, *p* < 0.01. Work engagement is positively and significantly related to Healthy Organisational Outcomes (i.e., in-role job performance, extra-role job performance, and affective organisational commitment), β = 0.71, *p* < 0.01, and has a partially mediating role between BPN satisfaction and Healthy Organisational Outcomes, β = 0.23, *p* < 0.05. Regarding BPN frustration (i.e., autonomy, relatedness, and competence), the results show a negative and non-significant relationship with work engagement, β = −0.17, *p* = −0.160, happiness, β = 0.005, *p* = 0.974, and Healthy Organisational Outcomes (i.e., in-role job performance, extra-role job performance, and organisational affective commitment), β = 0.12, *p* = 0.117. In the same way, happiness shows a non-significant relationship with Healthy Organisational Outcomes, β = 0.07, *p* = 0.071.

**Figure 4:**
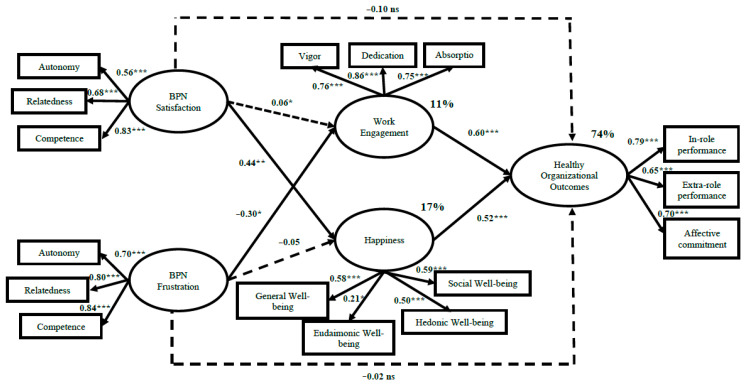
Final Model: Structural model of BPN, well-being, and Healthy Organisational Outcomes in men (N = 133). Notes: * *p* < 0.05; ** *p* < 0.01, *** *p* < 0.001.

Regarding the men’s group model (see Figure 4), happiness (i.e., general well-being, eudemonic well-being, hedonic well-being, and social well-being) fully mediates the relationship between BPN satisfaction (i.e., autonomy, relatedness, and competence) and Healthy Organisational Outcomes (i.e., in-role job performance, extra-role job performance, and organisational affective commitment). In addition, BPN satisfaction has a non-significant relationship with work engagement. Nevertheless, work engagement has a positive and significant relationship with Healthy Organisational Outcomes. The results show that BPN satisfaction (i.e., autonomy, relatedness, and competence) is positively and significantly linked to happiness (i.e., general well-being, eudemonic well-being, hedonic well-being, and social well-being), β = 0.44, *p* < 0.01, and has a non-significant relationship with work engagement (i.e., vigour, dedication, and absorption), β = 0.06, *p* = 0.646. In addition, work engagement is positively and significantly linked to Healthy Organisational Outcomes (i.e., in-role job performance, extra-role job performance, and affective organisational commitment), β = 0.60, *p* < 0.001. Happiness fully mediates the relationship between BPN satisfaction and Healthy Organisational Outcomes, β = −0.10, *p* = 0.461. Regarding BPN frustration (i.e., autonomy, relatedness, and competence), the results show a negative and significant relationship with work engagement, β = −0.30, *p* < 0.05, and a non-significant relationship with happiness, β = −0.05, *p* = 0.728, and Healthy Organisational Outcomes (i.e., in-role job performance, extra-role job performance, and organisational affective commitment), β = −0.02, *p* = 0.886. Furthermore, happiness shows a significant relationship with Healthy Organisational Outcomes, β = 0.52, *p* < 0.001. Regarding the Alternative Model (Mw/m), the results show a good data fit with regards to its absolute and relative indicators, χ^2^ (188) = 400.60, χ^2^/df = 2.13, RMSEA = 0.054, CFI = 0.91, NFI = 0.84, IFI = 0.91, AIC = 632.60, HOELTER = 218 (0.05) and 233 (0.01).

## 5. Discussion

This study contributes to our understanding of the relationship between the satisfaction/frustration of Basic Psychological Needs (BPN of autonomy, relatedness, and competence), well-being, and Healthy Organisational Outcomes within two Colombian food companies. Following Structural Equation Modelling (generally and according to the gender perspective), we tested the mediator role of well-being (i.e., engagement and happiness) in the relationship between PBN and Healthy Organisational Outcomes (in-role and extra-role job performance and organisational affective commitment) from a gender perspective.

Based on the Hypothesis, results show that Hypotheses 1 and 2 were partially confirmed. Regardless of the gender perspective, the results are in line with Ryan and Deci’s [26] proposal that the satisfaction of the need for autonomy, relatedness, and competence increases well-being, triggering an increase in self-motivation to meet challenges and goals in different areas (i.e., personal, organisational). Specifically, and with regards to WE, the results are consistent with previous research showing that, when people satisfy their needs for autonomy, relatedness, and competence, they increase their energy levels (i.e., vigour), feel pride and purpose in their tasks (i.e., dedication), and dedicate more hours to the tasks they enjoy, as well as those that require greater skill (i.e., absorption) [68]. These relationships have previously been demonstrated in the fields of education and sport [7,9,24]. The results of our study focus on the relevance of the satisfaction of BPN in the promotion of well-being in the work context. In addition, these results are in line with the large body of empirical evidence that supports the idea that self-determination positively influences organisational results [39]. It seems that the hypothesis that ‘a happy worker is a productive worker’, postulated by the Harvard Business Review [69], is fulfilled. This means that, when workers feel psychologically linked to their jobs in terms of WE (i.e., vigour, dedication, and absorption) and happiness (i.e., general well-being, eudemonic well-being, hedonic well-being, and social well-being), they will fulfil the tasks their role requires of them. Furthermore, they will perform proactive behaviours for the benefit of the organisation and the fulfilment of its goals, and they will have a strong intention to stay in the organisation [47,69]. In addition, in general terms, these results follow the postulates of the HERO Model [17,20], which indicates that the psychological well-being of workers, in terms of healthy employees, is a key element in explaining the relationship between resources (i.e., personal, work, social, and organisational) and Healthy Organisational Outcomes [70], regardless of gender. However, results by gender using SEM analyses show significant structural differences between men and women in the relationships among the model variables. Specifically, in the case of women, only work engagement plays a mediator role between BPN satisfaction and Healthy Organisational Outcomes. Moreover, only in women, a direct relationship is obtained between BPN satisfaction and Healthy Organisational Outcomes.

Hypotheses 3 and 4 were partially confirmed. Regardless of gender, BPN frustration was negatively and significantly related to WE, but it was not significantly related to happiness. These results are in line with those obtained in previous studies revealing that BPN frustration is related to high levels of stress and physical and psychological discomfort [25], which shows that frustration is not a determining factor in the more motivational aspects. It also appears that, when people feel frustrated in their BPN of autonomy, such factors as relatedness, competence, and motivational processes that make them psychosocially involved with their work tasks, are not activated. Hence, the negative relationship obtained in the study between BPN frustration and WE can harm the results of the in-role and extra-role job performance of workers, as well as their level of commitment. In contrast, unhealthy deterioration processes could be activated that would have the consequence of psychosocial discomfort at work, such as burnout syndrome. However, BPN frustration and happiness are not so determinant in the enhancement of performance and affective commitment in women. It seems that the frustration does not play any role in the reduction of engagement or happiness.

In contrast, the results for men are different. Specifically, results confirmed the full mediation of happiness among BPN satisfaction and Healthy Organisational Outcomes, as well the full mediation of work engagement among BPN frustration and Healthy Organisational Outcomes. Men with satisfaction of autonomy, relatedness, and competence show more happiness (but not work engagement), which, in turn, increases their Healthy Organisational Outcomes. Secondly, men with BPN frustration reduce their levels of work engagement, which, in turn, produces lower levels of Healthy Organisational Outcomes. In summary: while men’s happiness is determined by BPN satisfaction, its frustration only affects work engagement (but not happiness). For women, only BPN satisfaction plays a relevant role in the development of engagement and happiness, but only engagement is a drive (jointly with BPN satisfaction) for the development of performance and affective commitment. Consequently, Hypotheses 1 and 4 are partially confirmed, and Hypotheses 2 and 3 are confirmed in men.

## 6. Conclusions

In conclusion, our findings suggest the relevance of the Basic Psychological Needs for promoting well-being (engagement and happiness) and Healthy Organisational Outcomes within two Colombian food companies. Two different processes were obtained regardless of gender. The first is characterised by BPN satisfaction, which enhances well-being (work engagement and happiness) and leads to increasing the determinants of Healthy Organisational Outcomes. The second is related to BPN frustration, which only reduces levels of work engagement, which, in turn, reduces levels of Healthy Organisational Outcomes. Finally, relevant differences were obtained in these processes according to the gender perspective. Researchers and HR professionals must consider these relevant differences to guarantee well-being and Healthy Organisational Outcomes in both men and women.

### 6.1. Theoretical Implications

Theoretically, this study provides evidence about BPN satisfaction/frustration and its role in terms of psychological well-being and Healthy Organisational Outcomes, from a gender perspective, within two Colombian food companies. In addition, it provides evidence supporting the HERO Model, for the first time incorporating the role of BPN in its impact on people’s well-being (i.e., happiness and work engagement) and Healthy Organisational Outcomes (i.e., in-role and extra-role job performance and affective organisational commitment). In addition, the study introduces new elements of analysis into the business problem of well-being in organisations by applying the SDT [21,22] and the HERO Model [17,20] in the organisational area, given that their applied use has usually been undertaken in sports and education practices. This study also allows for the extension of both models according to a gender perspective, providing evidence of the differential impact of BPN in well-being and Healthy Organisational Outcomes for men and women.

Results of the ANOVA showed significant differences in the frustration of the need of autonomy and in organisational affective commitment, in favour of men. Finally, results of the SEM analyses with the whole sample, taking the gender perspective into account, partially confirmed our hypotheses. In particular, the results showed that, regardless of gender, psychological well-being (i.e., happiness and work engagement) fully mediates the relationship between BPN and Healthy Organisational Outcomes (i.e., in-role job performance, extra-role job performance, and organisational affective commitment). However, the relationship among the variables in the study is different for women and men. Particularly interesting is the relationship between BPN and psychological well-being according to gender, since BPN satisfaction generates a positive effect in well-being and Healthy Organisational Outcomes in women only.

### 6.2. Practical Implications

In practical terms, this study provides evidence about the Human Resources Management in both participating food organisations in Colombia, in terms of the relevant role of BPN satisfaction and frustration. HR professionals could use this information to conduct interventions that focus on optimising satisfaction of BPN and reducing frustration of BPN, taking the gender perspective into account. When workers perceive themselves as autonomous in carrying out their work tasks, consider that they have a reliable and healthy social climate at work, and perceive that their skills and abilities are constantly being enhanced, they will enter a positive underlying motivational process that will influence their psychological well-being and organisational results, thus generating Healthy Organisational Outcomes. Furthermore, particularly in women, BPN satisfaction should be addressed to guarantee well-being and positive results.

### 6.3. Limitations and Research Directions

The present study has some limitations. First, the study used a convenience sample, and only two Colombian food sector organisations participated. However, as 565 workers participated, this allows an organisational diagnosis of the variables measured. Second, it is a cross-sectional study, which does not allow conclusions to be drawn about the effect of the variables. However, it was possible to perform SEM analysis and evaluate the impact of an alternative model. In addition, we included measures with different scales (e.g., engagement was measured from 0 to 6, while happiness was measured using the original scale from 0 to 10). Despite this, there is evidence that the use of the different anchor points of a Likert sale does not represent a significant problem when performing analyses [71]. Fourth, this study integrates BPN satisfaction/frustration (autonomy, relationship, and competence), which provides evidence not only about the motivational process of BPN satisfaction, but also the role of frustration between psychological well-being and Healthy Organisational Outcomes. Finally, further studies need to be carried out to test the role of happiness, since some dimensions (particularly the eudemonic) showed low values. Despite this issue, we decided not to eliminate happiness from the model or the analyses, for several reasons: (1) this is the first time two dimensions of well-being were tested as mediators between BPN satisfaction/frustration, (2) by including work engagement and happiness we combined two ways to evaluate well-being within the same study: one in the work context (engagement) and the other one in a free context (happiness), (3) satisfaction accounts for 16%, 15%, and 17% of the variance in happiness in the total sample, in women and in men, respectively (as opposed to 13%, 14%, and 11% of the variance in work engagement), (4) happiness has a positive impact on Healthy Organisational Outcomes when the total sample is considered, as well as in men, and (5) the values of the dimensions of happiness are statistically significant (with the exception of the eudemonic dimension in the women’s sample).

Future research should, first, replicate research that considers both BPN satisfaction and frustration, contributing a double perspective, and study their role in the motivational process in organisations and well-being in companies from other sectors and cultures. In addition, this research should be extended to include other productive sectors to test the model analysed in this study. Finally, it would be interesting to consider longitudinal and quasi-experimental designs that can show the impact of interventions that promote BPN satisfaction over time.

## Figures and Tables

**Table 1:** Demographic information of the sample (age range, type of contract and tenure, N = 565).

Variables	Demographic Information
Age range	72% (408) aged 21–29; 19% (106) aged 30–39; 7% (30) aged 40–49; 2% (13) aged 50–60
Type of contract	92% (591) permanent contract; 3.7% (21) another types of contract; 2.1% (12) temporary contract
Tenure	57.5% (325) one to five years; 14% (56) six to 10 years; 4.4% (24); 21 to 32 years; 2.9% (16) 16 to 20 years; 2.2% (12) 11 to 15 years.

**Table 2:** Means, standard deviations, reliability, and intercorrelations among the study variables at (N = 565) as well as by gender.

Variables	Mean	Mean F	Mean M	SD	SD F	SD M	α	2	3	4	5	6	7	8	9	10	11	12	13
1. Autonomy satisfaction	4.94	5.00	4.82	1.22	1.15	1.23	0.70	0.51 ***	0.57 ***	−0.34 ***	−0.27 ***	−0.34 ***	0.23 ***	0.33 ***	0.33 ***	0.39 ***	0.30 ***	0.18 ***	0.34 ***
2. Relatedness satisfaction	5.19	5.27	5.14	1.10	1.04	1.03	0.70	-	0.64 ***	−0.35 ***	−0.43 ***	−0.35 ***	0.22 ***	0.28 ***	0.22 ***	0.31 ***	0.22 ***	0.20 ***	0.29 ***
3. Competence satisfaction	5.52	5.53	5.54	0.95	0.94	0.93	0.82		-	−0.34 ***	−0.46 ***	−0.45 ***	0.16 ***	0.23 ***	0.23 ***	0.35 ***	0.17 ***	0.16 ***	0.25 ***
4. Autonomy frustration	1.90	1.29	2.18	1.66	1.59	1.74	0.71			-	0.53 ***	0.59 ***	−0.33 ***	−0.40 ***	−0.27 ***	−0.25 ***	−0.24 ***	−0.14 ***	−0.21 ***
5. Relatedness frustration	1.35	1.29	1.39	1.44	1.48	1.45	0.70				-	0.63 ***	−0.19 ***	−0.21 ***	−0.14 ***	−0.21 ***	−0.18 ***	−0.14 ***	−0.15 ***
6. Competence frustration	1.21	1.18	1.32	1.40	1.46	1.34	0.70					-	−0.14 ***	−0.18 ***	−0.20 ***	−0.23 ***	−0.17 ***	−0.15 ***	−0.12 ***
7. Engagement: Vigor	4.56	4.57	4.48	1.01	0.98	1.10	0.84						-	0.66 ***	0.57 ***	0.26 ***	0.44 ***	0.36 ***	0.42 ***
8. Engagement: Dedication	4.77	4.77	4.79	1.04	1.08	1.03	0.81							-	0.66 ***	0.35 ***	0.45 ***	0.38 ***	0.52 ***
9. Engagement: Absorption	4.26	4.28	4.30	1.18	1.23	1.10	0.72				-				-	0.37 ***	0.33 ***	0.31 ***	0.51 ***
10. Happiness	8.12	8.15	7.91	1.40	1.36	1.56	0.83									-	0.27 ***	0.25 ***	0.38 ***
11. In-role performance	4.64	4.64	4.72	1.04	0.99	1.07	0.82										-	0.56 ***	0.44 ***
12. Extra-role performance	4.81	4.81	4.77	1.03	1.02	1.01	0.78											-	0.43 ***
13. Affective conmitment	4.70	4.83	4.53	1.25	1.08	1.11	0.79												-

Notes: SD = Standard deviation, α = Cronbach’s alpha, F = female, M = male, *** *p* < 0.001.

**Table 3:** Structural Equation Modelling (N = 565).

Model	χ^2^	gl	χ^2^/gl	*p*	RMSEA	CFI	NFI	IFI	AIC	HOELTER 0.01	HOELTER 0.05
M_1_	300.07	94	3.19	0.007	0.07	0.91	0.87	0.91	416.08	156	170
M_A_	467.31	96	4.87	0.000	0.09	0.83	0.80	0.84	579.31	102	112

Notes. M_1_ = Model 1, M_A_ = Alternative Model, χ^2^ = Chi-squared; gl = degrees of freedom; *p* = probability, RMSEA = Root Mean Square Error of Approximation; CFI = Comparative Fit Index; NFI = Normed Fit Index; IFI = Incremental Fit Index; AIC = Akaike information Criterion.
